# Carbon-Source Effects on Growth and Secondary Metabolism in the Marine Bacteroidota *Tenacibaculum mesophilum* and *Fulvivirga kasyanovii*

**DOI:** 10.3390/md23100394

**Published:** 2025-10-04

**Authors:** Luis Linares-Otoya, Virginia Linares-Otoya, Gladys Galliani-Huamanchumo, Terecita Carrion-Zavaleta, Jose Condor-Goytizolo, Till F. Schäberle, Mayar L. Ganoza-Yupanqui, Julio Campos-Florian

**Affiliations:** 1 Faculty of Pharmacy and Biochemistry, National University of Trujillo, Trujillo 13001, Peru; mlinares@unitru.edu.pe (V.L.-O.); ggalliani@unitru.edu.pe (G.G.-H.); terecita9307@gmail.com (T.C.-Z.); jcondor@unitru.edu.pe (J.C.-G.); mganoza@unitru.edu.pe (M.L.G.-Y.); 2 Institute for Insect Biotechnology, Justus-Liebig-University of Giessen, 35392 Giessen, Germany; till.f.schaeberle@agrar.uni-giessen.de; 3 Fraunhofer Institute for Molecular Biology and Applied Ecology, Branch for Bioresources, 35392 Giessen, Germany; 4 German Center for Infection Research (DZIF), Site Giessen/Marburg/Langen, 35390 Marburg, Germany; 5 Research Laboratory 1–Mixo (Pharmacology), Faculty of Pharmacy and Biochemistry, National University of Trujillo, Trujillo 13001, Peru

**Keywords:** Bacteroidota, biosynthetic gene clusters, secondary metabolite

## Abstract

Marine Bacteroidota are recognized bacterial producers of bioactive metabolites, yet their biosynthetic potential remains cryptic under standard laboratory conditions. Here, we developed chemically defined media for *Fulvivirga kasyanovii* 48LL (Cytophagia) and *Tenacibaculum mesophilum* fLL (Flavobacteriia) to evaluate the effect of environmentally relevant carbon sources on growth and secondary metabolism. *F. kasyanovii* utilized 31 of 34 tested carbon sources whereas *T. mesophilum* grew on only five substrates, underscoring a distinct nutritional preferences. Substrate significantly influenced the antibacterial activity of *F. kasyanovii* extracts. Growth on *β*-1,3-glucan, glycerol, poly(*β*-hydroxybutyrate) (PHB), fish gelatin, or pectin resulted in extracts generating the largest inhibition zones (10–13 mm) against *Bacillus subtilis* or *Rossellomorea marisflavi*. Genome analysis revealed *F. kasyanovii* to be enriched in biosynthetic gene clusters (BGCs), notably harboring a ~570 kb genomic island comprising five large NRPS/PKS-type clusters. Quantitative PCR confirmed carbon-source-dependent regulation of these operons: glucose induced BGC1, BGC3, and BGC4, while *κ*-carrageenan and PHB upregulated BGC2. Conversely, yeast–peptone medium (analogous to standard marine broth) repressed transcription across all active clusters. These findings demonstrate that naturally occurring carbon sources can selectively activate cryptic BGCs and modulate antibacterial activity in *F. kasyanovii*, suggesting that similar strategy can be used for natural-product discovery in marine Bacteroidota.

## 1. Introduction

The alarming rise of antimicrobial-resistant infections has renewed interest in mining unconventional ecological niches for chemically diverse antibiotics [1]. Marine microbiomes, in particular, have already yielded numerous structurally unique antibacterial scaffolds; yet comparative-genomic surveys indicate that their biosynthetic capacity is far from exhausted, because most secondary-metabolite biosynthetic gene clusters (BGCs) detected in marine bacterial genomes remain “orphan”, with no corresponding metabolite isolated to date [2].

Among the bacterial lineages that harbor large reservoirs of uncharacterized biosynthetic gene clusters (BGCs) are members of the phylum Bacteroidota [3]. These bacteria are historically recognized for playing key roles in coastal carbon cycling due to their high capacity to depolymerize algal glycans and other recalcitrant polymers [4]. However, in recent years, large-scale comparative-genomic surveys have revealed that marine Bacteroidota encode hundreds of mostly novel secondary-metabolite BGCs whose products remain unexplored [5]. In that study, the authors catalogued 2594 BGCs (1–22 BGCs per strain) from 306 structural families, but only 12 families were linked to known metabolites, indicating that most clusters probably encode undiscovered chemistry [5]. The chemical potential of these clusters is evidenced by several antibacterial compounds isolated from Bacteroidota species, for instance, the hybrid polyketide-peptide ariakemicins A–B from a *Rapidithrix* sp. [6], the chlorinated peptides aquimarins A–C from *Aquimarina* sp. Aq135 [7], and the 16-membered macrolides YM-32890 A–B [8] from *Cytophaga* sp. YL-02905S. Nevertheless, the group remains strikingly under-represented in antibiotic-discovery pipelines, which still prioritize traditional producer phyla such as Actinobacteria and Proteobacteria [3].

A plausible explanation for the scarcity of Bacteroidota-derived antibiotics is that many of their biosynthetic gene clusters (BGCs) remain transcriptionally silent under routine laboratory conditions that do not reproduce the ecological cues controlling gene expression in situ [9]. Because marine Bacteroidota specialize in polysaccharide catabolism, an ability encoded by expansive polysaccharide-utilization loci (PULs) [10], we hypothesized that algal or other naturally occurring carbohydrates might act as physiological triggers for cryptic BGC activation. The “One Strain–Many Compounds” (OSMAC) strategy, which induces the production of secondary metabolites through systematic medium optimization, substantiates this premise: even subtle changes to carbon-source composition have repeatedly unleashed hidden metabolites in actinomycetes and other model producers [11]. In that sense, previous studies used chemically defined media to show that switching carbon sources can strongly induce or repress secondary-metabolite BGCs, and that the resulting expression patterns vary even at the intra-species level [12]. Yet, similarly substrate-resolved studies that couple nutrient preference to BGC transcription and metabolite output in marine Bacteroidota have not been systematically pursued.

Here, we tackle this knowledge gap by examining two phylogenetically distant marine Bacteroidota isolated from the Peruvian intertidal zone: *Fulvivirga kasyanovii* 48LL (class Cytophagia) and *Tenacibaculum mesophilum* fLL (class Flavobacteriia). We first formulated chemically defined media that enabled systematic testing of a broad panel of carbon sources, including low-molecular-weight compounds, polymeric polysaccharides, and complex peptidic substrates. Using these media, we quantified carbon-dependent antibacterial activity in culture extracts of *F. kasyanovii*. In parallel, we sequenced the 7.5 Mb genome of *F. kasyanovii*, mapped its biosynthetic repertoire, and used qPCR to track the transcriptional response of individual biosynthetic gene clusters (BGCs) to selected carbon sources. This integrated physiological, bioactivity-based, and transcriptomic analysis provides a rational framework for future natural-product prospecting in this underexplored phylum.

## 2. Results

### 2.1. Carbon Source Preferences of Fulvivirga kasyanovii 48LL and Tenacibaculum mesophilum fLL in Defined Media

To investigate whether secondary metabolism in marine Bacteroidota can be regulated by environmentally relevant carbon sources, we first developed a chemically defined medium that allows the response to individual substrates to be tested. We focused on two taxonomically distant Bacteroidota strains that we previously isolated from the intertidal zone in Peru: *F. kasyanovii* 48LL (Class Cytophagia) and *T. mesophilum* fLL (Class Flavobacteriia) [13,14], for which no defined media had been previously reported. For *F. kasyanovii*, we initially conducted growth tests in 75% artificial seawater (ASW) using glucose (0.5 g L ^−1^) as the carbon source, and either ammonium phosphate (2 g L^−1^) or a defined amino acid mixture (arginine 125 mg L^−1^, lysine 125 mg L^−1^, glycine 250 mg L^−1^, cysteine 62.5 mg L^−1^) as nitrogen sources. Both nitrogen sources supported growth, but the amino acid mixture resulted in qualitatively superior growth. Optimization of ferrous sulfate concentration (5–100 mg L^−1^) identified 100 mg L^−1^ as optimal, and this formulation was used for subsequent carbon source preference assays, in which we replaced glucose by alternative substrates. In contrast, *T. mesophilum* fLL did not grow in 75% ASW with glucose in combination with various nitrogen sources (amino acids or ammonium phosphate, with or without ferrous sulfate). Suspecting that additional or alternative carbon sources were required, we broadened the screening to include a mixture of 22 amino acids (Table A1 and Table A2), which successfully supported modest growth. Systematic simplification of this mixture led to the development of a minimal defined medium containing 10 amino acids, which was used in subsequent experiments (Section 4).

**Figure 1:**
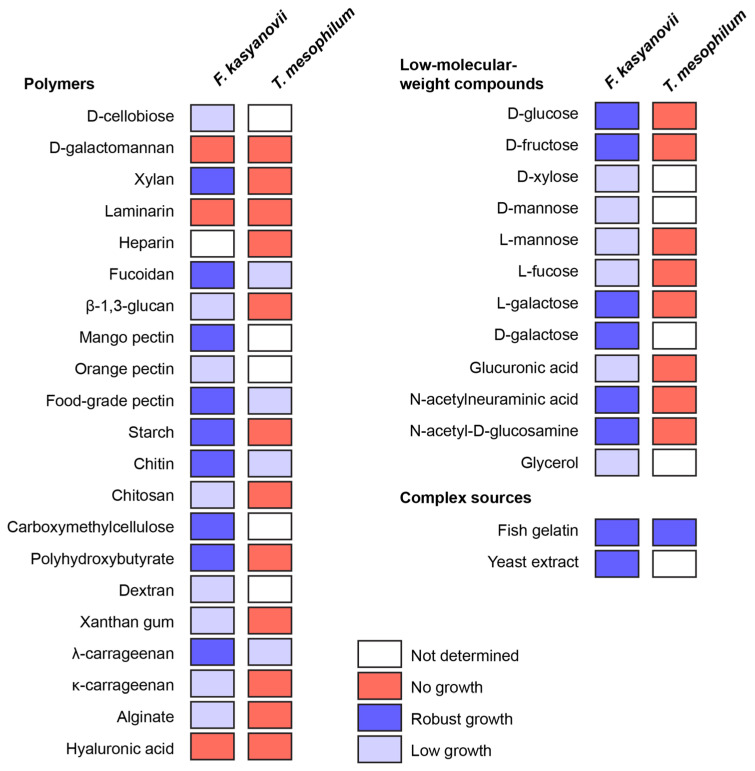
Utilization of low-molecular-weight, polymeric, and complex carbon sources by *F. kasyanovii* and *T. mesophilum*. Squares indicate growth after 6 days in the respective defined media (30 °C, 180 rpm) containing a single substrate. Growth was qualitatively assessed (robust growth: blue, low growth: lavender, no growth: red, not determined: white) and viability of the cultures was confirmed by inoculating culture in standard marine agar. Substrates are grouped by chemical class: polysaccharide or polymeric molecules (**left**), monosaccharides and other low-molecular-weight compounds (**right top**), and complex nutrient sources (**right bottom**).

Then, using the developed defined media, we tested growth of *F. kasyanovii* (34 carbon sources) and *T. mesophilum* (25 carbon sources) in 6-day shake-flask incubations (30 °C, 130 rpm; Figure 1). The substrate panel (Table A3) included low-molecular-weight compounds (D-glucose, D-fructose, D-xylose, D-mannose, L-mannose, L-fucose, L-galactose, D-galactose, L-fructose, glucuronic acid, N-acetylneuraminic acid, N-acetyl-D-glucosamine, glycerol). Additionally, natural (most reported from marine habitats) and synthetic polymers included D-cellobiose, D-galactomannan, xylan, laminarin, heparin, fucoidan, mango pectin, orange pectin, food-grade pectin, starch, chitin, chitosan, carboxymethylcellulose, dextran, xanthan gum, *λ*-carrageenan, *κ*-carrageenan, alginate, hyaluronic acid, *β*-1,3-glucan, and poly *β*-hydroxybutyrate. Complex peptidic sources used were yeast extract and gelatin from cold-water fish skin. We observed that *T. mesophilum* exhibited a narrow substrate utilization profile, growing on only 5 of the 25 tested substrates: food-grade pectin, *λ*-carrageenan, fucoidan, fish skin gelatin, and chitin (Figure 1). Among these, we only observed robust growth on the medium that was supplemented with fish skin gelatin. In contrast, *F. kasyanovii* utilized 31 of the 34 tested substrates, failing to grow only on laminarin, galactomannan, and hyaluronic acid (Figure 1). Growth varied significantly across substrates: robust growth occurred on 17 substrates, while 14 supported only modest to low growth (D-xylose, D-mannose, L-mannose, L-fucose, glucuronic acid, glycerol, cellobiose, orange pectin, chitosan, carboxymethylcellulose, dextran, xanthan gum, *κ*-carrageenan, alginate, and *β*-1,3-glucan). Collectively, these results indicate a stark contrast in substrate utilization profile between the two strains, with *F*. *kasyanovii* utilizing a broader range of carbon sources than *T*. *mesophilum*.

### 2.2. Carbon-Source-Dependent Induction of Antimicrobial Activity of F. kasyanovii Extracts

**Figure 2:**
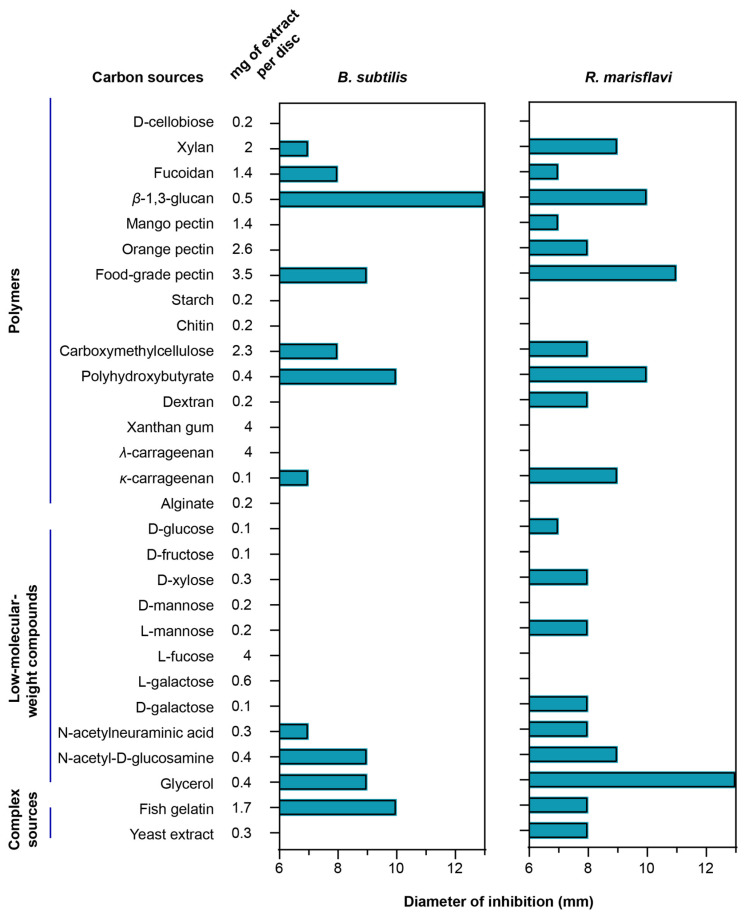
Carbon-source-dependent antibacterial spectrum of *F. kasyanovii* culture extracts. A bar graph indicating the measured inhibition-zone diameter (mm) produced by chemical extracts of *F. kasyanovii* cultures against *B. subtilis* ATCC 6633 (**left**), *R. marisflavi* sv176 (**right**) including the 6 mm disc. Carbon sources are organized by chemical class: polymeric carbohydrates, low-molecular-weight compounds, and complex nutrient sources. Chemical extracts prepared from 7-day cultures grown in defined media supplemented with the indicated carbon sources (first column) were applied to 6 mm paper discs at the concentrations indicated in the second column.

To evaluate how different carbon sources influence secondary-metabolite production, we compared the antibacterial activity of extracts obtained from *F. kasyanovii* cultures grown on diverse substrates. The same analysis was not feasible for *T. mesophilum*, as this strain grew robustly on only one carbon source, precluding comparative analysis. We cultured *F. kasyanovii* for 7 days at 30 °C with agitation (130 rpm) in a defined medium individually supplemented with 29 of the growth-supporting carbon sources (Figure 2). Then, we chemically extracted the cell-free supernatants on reversed-phase C18 resin to yield crude metabolite extracts. These extracts were screened in standard disk-diffusion assays against two Bacillales representatives, *Bacillus subtilis* ATCC 6633 and the marine isolate *Rossellomorea marisflavi* sv176 (Figure 2). As positive controls for *B. subtilis* and *R. marisflavi,* we used ampicillin (0.375 µg) and erythromycin (0.833 µg), respectively, while solvent-only and blank-medium extracts were included as negative controls.

Of the 29 extracts tested, 11 substrates produced measurable inhibition zones (7–13 mm) against *B. subtilis* (xylan, fucoidan, *β*-1,3-glucan, food-grade pectin, carboxymethyl-cellulose, polyhydroxybutyrate, *κ*-carrageenan, N-acetyl-neuraminic acid, N-acetyl-d-glucosamine, glycerol, and fish gelatin). Against *R. marisflavi,* the same carbon sources plus eight additional substrates (mango pectin, orange pectin, dextran, d-glucose, d-xylose, l-mannose, d-galactose, and yeast extract) showed antimicrobial activity, with inhibition zones ranging from 7 to 13 mm (Figure 2). The highest activity (10–13 mm) against *B. subtilis* was observed for extracts from cultures supplemented with *β*-1,3-glucan, polyhydroxybutyrate, and fish gelatin, while the strongest bioactivity against *R. marisflavi* (10–13 mm) was obtained for extracts from food-grade pectin, *β*-1,3-glucan, polyhydroxybutyrate, and glycerol supplemented media. As expected, no inhibition was observed in the negative controls, whereas the positive controls produced prominent inhibition zones (ampicillin: 18 mm; erythromycin 23 mm). Together, our results indicate that the choice of carbon source can differentially influence the production of bioactive secondary metabolites, as reflected in the observed distinct antibacterial profiles.

### 2.3. Secondary Metabolite Biosynthetic Gene Clusters of F. kasyanovii 

**Figure 3:**
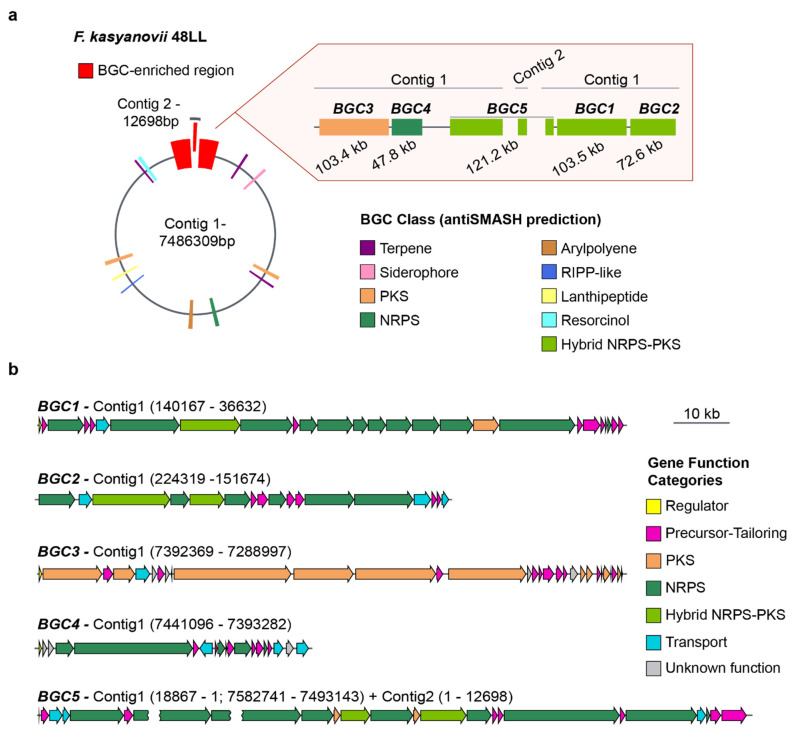
Secondary metabolite biosynthetic gene clusters in *F. kasyanovii* 48LL. (**a**) Representation of the 7.49 Mb genome (contig 1, 7.486 Mb; contig 2, 12.7 kb). Colored boxes mark the 16 secondary-metabolite BGCs predicted by antiSMASH; those within a ~570 kb BGC-enriched region (red) are enlarged at right. The expanded view shows the order, length, and predicted class of BGC1–BGC5: BGC3 (103.4 kb, PKS), BGC4 (47.8 kb, NRPS), BGC5 (121.2 kb, hybrid NRPS-PKS; spans the contig-junction), BGC1 (103.5 kb, hybrid NRPS-PKS), and BGC2 (72.6 kb, NRPS). Additional classes present in the genome include terpenes, arylpolyenes, RiPP-like, lanthipeptide, siderophore, and resorcinol clusters (color legend). (**b**) Gene-level architecture of BGC1–BGC5. Arrows denote open reading frames drawn to scale and colored by predicted functional category: regulator (yellow), precursor/tailoring enzyme (magenta), polyketide synthase (orange), non-ribosomal peptide synthetase (green), hybrid NRPS-PKS (dark green), transport (cyan), and hypothetical/unknown (grey). The broken arrow in BGC5 indicates the contig break (contig 1 → contig 2). Scale bar, 10 kb.

**Figure A1:**
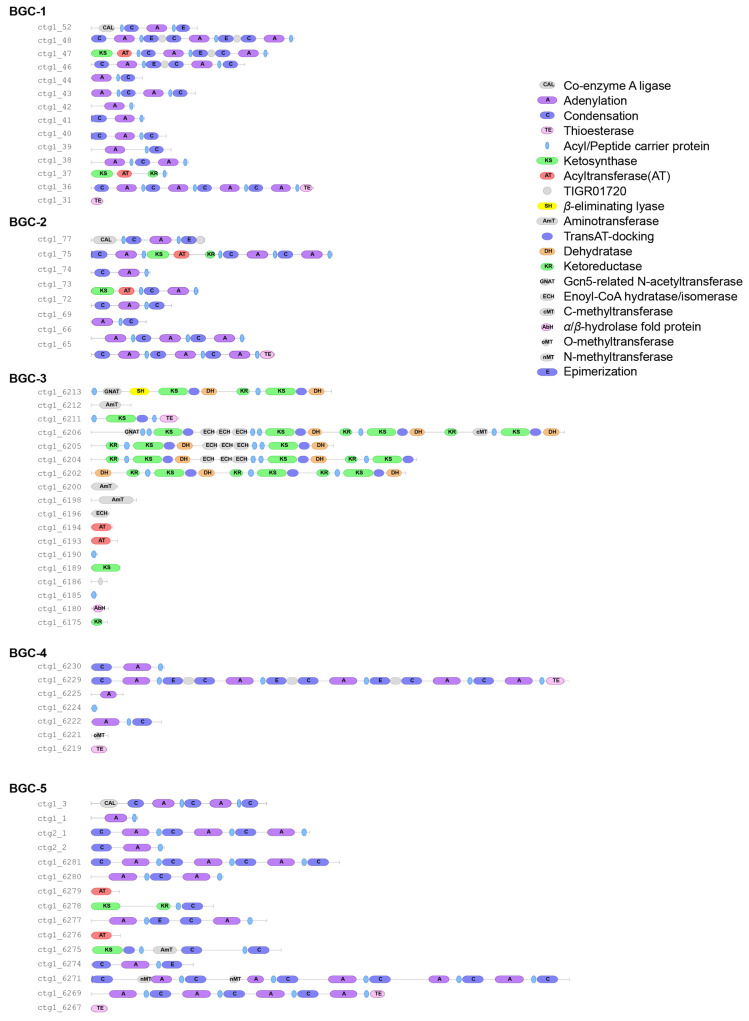
Domain organization of NRPS/PKS synthases from BGCs 1-5 using antiSMASH 8.0.

The broad, carbon-source-dependent bioactivities we observed suggested that the tested strains harbor multiple secondary-metabolite pathways. To explore this, we selected *F. kasyanovii* for whole-genome sequencing using Oxford Nanopore technology. We obtained a 7.49 Mb assembly that resolves into a primary 7.48 Mb contig and a secondary 13 kb contig (Figure 3a). Running antiSMASH v8.0 on this assembly [15], we identified 16 biosynthetic gene clusters (BGCs) spanning 11 biosynthetic classes and occupying approximately 10% of the genome. Notably, five of these BGCs co-localized into a single ~570 kb genomic region (~7.5% of the chromosome). This region contains three large hybrid non-ribosomal peptide synthetase/polyketide synthase (NRPS/PKS) clusters, BGC1 (103.5 kb), BGC2 (72.6 kb), and the partially resolved BGC5 (~121 kb), as well as a trans-AT PKS (BGC3, 103.4 kb) and NRPS cluster (BGC4, 47.8 kb). This BGC-enriched region is localized at both ends of the main contig, where the presence of repetitive sequences in BGC5 prevented complete closure, with part of this cluster extending into the 13 kb contig (Figure 3a, Figure A1). Besides encoding the typical large multimodular assembly enzymes, this BGCs-enriched region harbors a large number of genes encoding secondary metabolite precursor-biosynthesis and tailoring enzymes (37 genes) as well as transport proteins (11 genes), regulatory proteins (3 genes), and others with unknown function (8 genes) (Figure 3b).

The remaining BGCs were scattered across the chromosome and comprise three terpene clusters, one resorcinol cluster, one siderophore cluster, one arylpolyene cluster, one type I PKS, one type III PKS, two ribosomally synthesized and post-translationally modified peptide (RiPP) clusters, and one NRPS-like cluster (Figure 3a). Sequence similarity and synteny analyses against the MIBiG database revealed close homologues for only two clusters: an arylpolyene BGC, matching to flexirubin pathway (BGC0000838), and a siderophore BGC matching fulvivirgamide (BGC0002620.2). The remaining 14 clusters lack characterized counterparts, underscoring *F. kasyanovii* as a rich source of previously uncharacterized secondary metabolites.

### 2.4. Carbon-Source-Dependent Regulation of Biosynthetic Gene Clusters in F. kasyanovii

With the full *F. kasyanovii* genome sequence available, we set out to determine directly whether the nature of the carbon source modulates transcription of the biosynthetic gene clusters (BGCs) that we had identified bioinformatically. We focused on the five BGCs clusters (BGC1–BGC5) that co-localize within the BGC-enriched region. We wondered whether these BGCs, given their proximity in the chromosome, were coregulated, or if the individual clusters respond differentially to distinct nutritional cues. To quantify transcription, we used quantitative PCR (qPCR) to determine the expression of representative, non-repetitive regions within each cluster. Transcript abundance was normalized to the 16S rRNA reference gene and expressed as 2^-ΔCt [16]. We quantified gene expression after culturing *F. kasyanovii* for 48 h in a defined medium that was individually supplemented with seven carbon sources: four small molecules (glucose, N-acetyl-d-glucosamine, glycerol, and l-lysine) and three polymers (*κ*-carrageenan, poly(*β*-hydroxybutyrate) (PHB), and *β*-1,3-glucan). In addition, we included a complex yeast–peptone medium that is used as standard medium to cultivate marine bacteria. Expression varied markedly across clusters and media (*p* < 0.0001). Averaged over all conditions, BGC3 displayed the highest abundance (mean 2^-ΔCt = 8.9 × 10^−5^), followed by BGC4 (7.1 × 10^−5^), BGC1 (1.9 × 10^−5^), and BGC2 (2.2 × 10^−6^); BGC5 transcripts were negligible (3.7 × 10^−8^).

**Figure 4:**
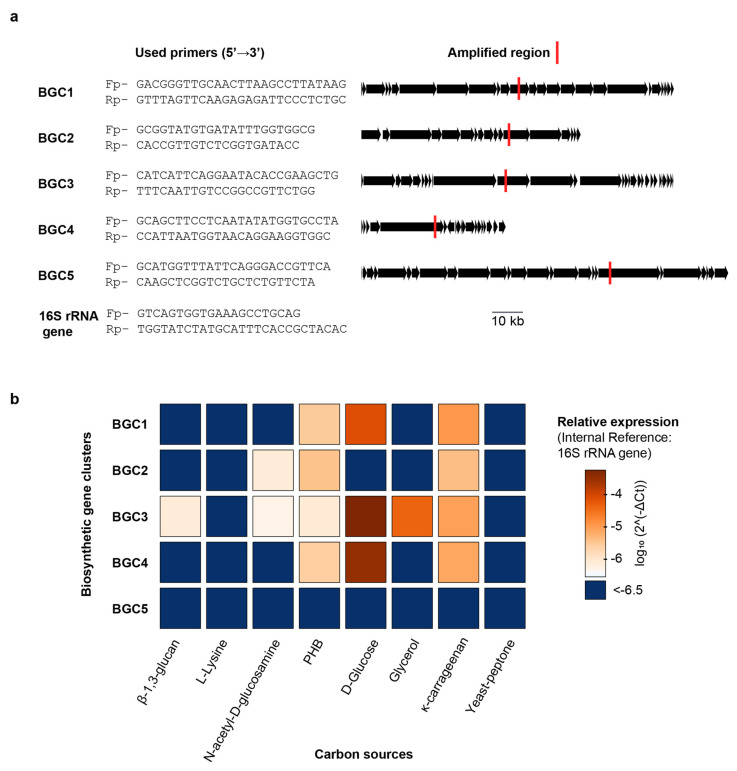
Carbon-source-dependent regulation of BGC1–BGC5 transcription in *F. kasyanovii***.** (**a**) Gene-specific primer pairs (5′→3′) designed for quantitative RT-PCR are listed on the left; the corresponding amplicons are mapped (red bars) onto linear representations of BGC1–BGC5 and the 16S rRNA reference gene. Additional information is summarized in Table A4 and Table A5. Black arrows denote open reading frames; the scale bar applies to all clusters. (**b**) Heat map of relative transcript abundance (log_10_ 2^-ΔCt, normalized to 16S rRNA) for the five BGCs after 48 h growth in defined medium (30 °C, 130 rpm) containing a single carbon source (*β*-1,3-glucan, L-lysine, N-acetyl-d-glucosamine, poly-*β*-hydroxybutyrate (PHB), d-glucose, glycerol, or *κ*-carrageenan) or a complex yeast/peptone medium. Color range indicates expression; dark blue indicates values below −6.5.

Despite being contiguous, clusters were not coregulated but, instead, they differentially responded to the carbon sources tested (Figure 4). BGC3 exhibited the highest expression levels in defined medium supplemented with glucose (2^-ΔCt = 5.6 × 10^−4^), and a significant decrease in complex media such as yeast–peptone, where expression dropped to 3.2 × 10^−7^, corresponding to a ~1740-fold reduction (*p* < 0.001). BGC3 expression was also significantly lower in glycerol, *κ*-carrageenan, *β*-1,3-glucan, N-acetyl-D-glucosamine, and PHB supplemented media but these conditions were still higher than yeast–peptone and L-lysine (Tukey *p* < 0.01). Interestingly, BGC3 was the only BGC induced by *β*-1,3-glucan and glycerol. Expression of BGC4 followed a similar pattern, with highest expression in glucose medium (3.5 × 10^−4^) and a ~1993-fold decrease in yeast–peptone medium (1.7 × 10^−7^; *p* < 0.001). Other carbon sources that induced BGC4 expression were PHB and *κ*-carrageenan, however at lower levels than glucose. Expression of BGC1 was also maximal in glucose medium (7.8 × 10^−5^) and significantly lower in yeast–peptone (1.8 × 10^−7^), with a fold change of ~442 (*p* < 0.001). Weaker but statistically significant higher expressions were observed for BGC1 in medium containing *κ*-carrageenan and poly(*β*-hydroxybutyrate), relative to yeast–peptone and L-lysine containing media.

BGC2 displayed a distinct expression profile (Figure 4), with the highest expression observed in medium with *κ*-carrageenan (2^-ΔCt = 4.2 × 10^−6^) and PHB (3.7 × 10^−6^), both with a ~30-fold increase over yeast–peptone (*p* < 0.0001). N-acetyl-d-glucosamine stimulated BGC2 less strongly (~5-fold lower than *κ*-carrageenan or PHB), yet still more than the remaining media (*p* < 0.01).

Taken together, our results reveal that naturally occurring carbon sources exert differential control over secondary-metabolite gene clusters, despite being located in the same chromosomal region. Thus, glucose was the strongest inducer of BGC1, BGC3, and BGC4 expression, whereas *κ*-carrageenan and PHB upregulated BGC2. In stark contrast, BGC5 is transcriptionally silent across the entire panel. Importantly, cultivation in yeast–peptone, analogous to commonly used marine broth medium, suppresses the expression of all four active clusters.

## 3. Discussion

In this study, we show how varying carbon sources in the media can result in differential regulation and production of secondary metabolites by a representative marine Bacteroidota strain. By formulating chemically defined minimal media and supplying single substrates, we systematically compared the growth of the intertidal isolates *F. kasyanovii* 48LL and *T. mesophilum* fLL and, for the former, describe how secondary metabolite profiles vary across conditions. The two species displayed sharply contrasting carbon-source preferences: *F. kasyanovii* utilized 31 of 34 test compounds, whereas *T. mesophilum* utilized only 5 of 25. Comparative genomics of their polysaccharide-utilization loci (PULs) suggest a mechanistic rationale. The *F. kasyanovii* genome encodes 17 PULs, exceeding the 11 found in *T. mesophilum* (Table A6), and most of its PULs resemble systems that degrade abundant marine polymers such as laminarin *β*-glucans, homogalacturonan pectins, and sulfated carrageenans [17]. By contrast, the substrates targeted by most *T. mesophilum* PULs remain unknown. Thus, the size and composition of the *F. kasyanovii* PUL repertoire are consistent with its broader substrate-utilization profile observed experimentally.

In *F. kasyanovii*, antibacterial activity was highly substrate-selective. Only eleven carbon sources produced extracts that inhibited *B. subtilis*, and a slightly broader, but still defined, subset was active against the marine bacillale *R. marisflavi*. Several of the strongest inducers, *β*-1,3-glucan, *κ*-carrageenan, glycerol, poly(*β*-hydroxybutyrate) (PHB), and fish gelatin, are abundant in the strain’s intertidal habitat [10], implying that specialized metabolites could be triggered by ecologically relevant cues rather than by general nutrient availability. However, it is worth noting that identifying the precise carbon sources relevant to a bacterium’s micro-habitat remains a considerable challenge. These findings provide support for the “One Strain–Many Compounds” (OSMAC) strategy where altering culture conditions can elicit different metabolites from the same microorganism [11]. Our findings extend OSMAC beyond its traditional actinobacterial and firmicute focus to marine Bacteroidota, underscoring the choice of ecologically relevant carbon sources as a practical lever for activating silent pathways. Indeed, there are several precedents of Bacteroidota producing secondary metabolites when non-conventional polysaccharide-rich carbon sources were used. For instance, *Flexibacter* sp. strain 758 required a medium with corn steep liquor and a high concentration of starch (4.8%) as only nutrient source to yield the potent elastase-inhibiting antibiotic FR901451 [18]. Another *Flexibacter* strain *F*. sp. SC 11479 produced the betalactamase-inhibitor monobactam SQ 28,502 only in a medium where tomato paste and oat flour were the sole carbon–nitrogen sources [19]. In *Empedobacter* sp., the cyclic depsipeptide empedopeptin (BMY-28117) was obtained using a linseed (flaxseed) meal as a carbon/nitrogen source along with sucrose [20]. Collectively, these examples and our data show that non-conventional, polysaccharide-rich substrates are powerful, yet underused, inducers of secondary-metabolite biosynthesis in Bacteroidota.

Our operon-level transcript analysis of *F. kasyanovii* confirms that the carbon-source-dependent effect on the secondary metabolism occurs at the transcriptional level. Within a 570 kb genomic island, five contiguous NRPS/PKS biosynthetic gene clusters (BGC1–BGC5) responded differently to the eight substrates tested. Glucose maximally induced BGC1, BGC3, and BGC4; *κ*-carrageenan and poly(*β*-hydroxybutyrate) preferentially activated BGC2; whereas BGC5 remained silent under all conditions. Such functional uncoupling among neighboring clusters implies dedicated sensing and regulatory circuits for distinct environmental cues. Similar substrate-specific transcriptional responses to complex polymers have been reported in other Bacteroidota [21,22], supporting a shared regulatory paradigm. Strikingly, in *F. kasyanovii*, the complex nutrient-rich yeast–peptone medium repressed tested biosynthetic gene clusters that were activated by at least one defined carbon source, mirroring observations in *Pedobacter lusitanus*, where casein peptone components suppress both BGC transcription and pedopeptin production [23]. Together, these data could explain why Bacteroidota sometimes appear “silent” in standard laboratory media. We envision that this work, while preliminary, represents an initial important step towards discovery novel metabolites from this unexplored strain, first by indicating that specific ecological carbon sources can de-repress these cryptic BGCs, therefore providing a guide to focus on certain growth conditions when attempting to isolate compounds. Second, our current work identifies which gene clusters would be feasible to target in later isolation efforts. Future work should focus on identifying the chemical products of these BGCs and determining whether the observed bioactivity is linked to these metabolites (e.g., using mass spectrometry, NMR, and synthetic biology). Overall, our results suggest that supplying ecologically relevant carbon sources would become an effective strategy for expressing the cryptic biosynthetic potential of marine Bacteroidota.

## 4. Materials and Methods

### 4.1. Cultivation of Bacterial Strains and Maintenance 

Working cryostocks *F. kasyanovii* 48LL and *T. mesophilum* fLL, previously isolated from marine sediments [13,14], were reactivated on marine agar 2216 at 30 °C for 24–48 h before each experiment. For defined media, artificial seawater was used as basal salt solution. A 5× ASW concentrate (pH 8.0) was prepared from NaCl 117.4 g L^−1^, KCl 3.3 g L^−1^, CaCl_2_·2H_2_O 7.35 g L^−1^, MgSO_4_·7H_2_O 53.05 g L^−1^, KBr 0.5 g L^−1^, NaHCO_3_ 0.95 g L^−1^, SrCl_2_·6H_2_O 0.20 g L^−1^, Na_2_SO_4_ 19.6 g L^−1^, and H_3_BO_3_ 0.15 g L^−1^, autoclaved (121 °C, 20 min) and diluted to 0.75× (*v*/*v*) with ultrapure water; 1 g L^−1^ CaCl_2_·2H_2_O was added after autoclaving.

### 4.2. Development of a Defined Medium for F. kasyanoviii 

First, 10 mL cultures (125 mL baffled flasks) received either NH_4_H_2_PO_4_ (2 g L ^−1^) or a 100 µL amino acid stock solution (1:1:1:1 vol, arginine 50 mg mL^−1^, lysine 50 mg mL^−1^, glycine 100 mg mL^−1^, cysteine 25 mg mL^−1^) as nitrogen sources and glucose as carbon source (500 mg mL^−1^). Growth after 48 h favored the amino acid formulation, which was later used for further experiments. FeSO_4_·7H_2_O was then titrated (5–100 mg L^−1^); optimal growth occurred at 0.10 g L^−1^, and this concentration was included in the defined media.

### 4.3. Development of a Defined Medium for T. mesophilum

Given that *T. mesophilum* was unable to grow in ASW + glucose + NH_4_H_2_PO_4_ or the 4-aminoacid solution tested for *F. kasyanovii*, a twenty-two amino acids solution was tested and resulted in growth (Table A1 and Table A2). We later iteratively reduced the number of amino acids and finally selected a medium where 10 mL of ASW was supplemented with 100 µL of a ten amino acids solution (50 mg mL^−1^, 9.1 µL glycine, 9.1 µL arginine, 9.1 µL lysine, 9.1 µL cysteine, 9.1 µL glutamate, 9.1 µL hydroxy-proline, 7.3 µL alanine, 7.3 µL proline, 7.3 µL aspartate, 5.8 µL serine).

### 4.4. Growth Test in a Carbon Source Library

Thirty-four substrates were separately sterilized and added to the defined media as specified in Table A3. Carbon sources included low-molecular-weight molecules such as d-glucose, d-fructose, d-xylose, l-mannose, l-fucose, l-galactose, l-fructose, d-glucuronic acid, N-acetyl-neuraminic acid, N-acetyl-d-glucosamine, and glycerol. Polymers and complex substrates included d-cellobiose, d-galactomannan, xylan, laminarin, heparin, fucoidan, mango pectin, orange pectin, food-grade pectin, starch, chitin, chitosan, carboxymethyl-cellulose, dextran, xanthan gum, *λ*-carrageenan, *κ*-carrageenan, sodium alginate, hyaluronic acid, *β*-1,3-glucan, and poly-*β*-hydroxybutyrate. Complex sources used were yeast extract and cold-water fish gelatin. Cells from *F. kasyanovii* or *T. mesophilum* overnight inoculum (obtained from 100 µL of MB culture) were washed with sterile deionized water and the cells were used to inoculate 10 mL of the defined medium with the selected carbon source. Bacteria were cultured at 30 °C, 130 rpm, for 6 days.

### 4.5. Chemical Extraction

Whole 7-day 120 mL cultures were sonicated (DC150H ultrasonic water bath, MRC Ltd. Holon, Israel) for 45 min, centrifuged (5000× *g*, 15 min, 4 °C) and filtered (0.45 µm). Supernatants were loaded onto 3 mL C_18_-SPE cartridges pre-equilibrated with 100% acetonitrile (ACN) followed by 5% ACN+0.1% formic acid (FA). After washing with two cartridge volumes of 5% ACN+0.1%FA, metabolites were eluted with 2 vol of 10% ACN+0.1%FA, 20% ACN+0.1%FA, 25% ACN+0.1%FA, 30% ACN+0.1%FA, 50% ACN+0.1%FA, 75% ACN+0.1%FA, and 100% ACN. All eluates were combined, evaporated at 40 °C (rotary evaporator), and re-dissolved in 0.5 mL 50% ACN+0.1%FA.

### 4.6. Antibacterial Assay of F. kasyanovii Chemical Extracts

Kirby–Bauer disc diffusion was performed on LB agar (*Bacillus subtilis* ATCC 6633) or marine agar (*Rossellomorea marisflavi* sv176). Paper discs (6 mm) received 20–40 µL extract (0.1–4 mg disc^−1^); controls were ampicillin (0.375 µg for *B. subtilis*) or erythromycin (0.833 µg for *R. marisflavi*), and blank media extracts and solvent only were negative controls. Plates were incubated for 16 to 24 h at 30 °C and inhibition halos were measured.

### 4.7. Genome Sequencing and BGC Characterization of F. kasyanovii

Whole-genome sequencing was performed by Plasmidsaurus Inc. (Watterson Park, KY, USA) using Oxford Nanopore long-read technology, and assemblies were generated with Flye and polished with Medaka. Biosynthetic gene clusters (BGCs) were identified with antiSMASH v8.0.1 using default settings [15], enabling comprehensive detection of terpene, siderophore, resorcinol, lanthipeptide, PKS, NRPS, RiPP, and hybrid clusters across the genome. Manual annotation of protein functionality and BGC boundaries was carried out for BGC 1–5, taking into account the functional annotation of the proteins, gene directionality, and intergenic spacing. For BGC5, NRPS-domain collinearity analysis was used.

### 4.8. RNA Extraction

Total RNA was obtained from specified 2-day bacterial culture (defined medium with glucose, N-acetyl-d-glucosamine, glycerol, l-lysine, *κ*-carrageenan, poly-(*β*-hydroxybutyrate, *β*-1,3-glucan, and yeast–peptone) using the PureLink™ RNA Mini Kit. Procedures were performed in a UV-irradiated, RNase-free workspace. An on-column DNase treatment (PureLink™ DNase, Waltham, MA, USA) was included. RNA quality was checked by agarose electrophoresis and quantified spectrophotometrically.

### 4.9. Reverse Transcription

cDNA synthesis was carried out using the GoScript™ Reverse Transcription System (Promega, Madison, WI, USA) with 60 µM Random Primers Mix (NEB). For each 20 µL reaction: RNA and primers were mixed and denatured at 70 °C; reverse-transcriptase mix (including RNase inhibitor, MgCl, nucleotides and water) was added; primer alignment was 25 °C; elongation 42 °C. Finally, quality was checked by electrophoresis.

### 4.10. Quantitative PCR

Quantitative PCR was used to measure gene expression of BGC1–5. Primers were designed to target non-repetitive regions in central regions of selected gene clusters. PCR reactions (10 µL) contained 5 µL of SsoAdvanced Universal SYBR Green Supermix, 0.125 µL each primer (10 µM), and 4.8 µL 1:10-diluted cDNA. Cluster-specific primer pairs (~100 bp amplicons) were validated for 87–102% efficiency (Table A5). Transcript abundance was normalized to the 16S rRNA reference gene and expressed as 2^-ΔCt [16] using the CFX Manager™ software (version 3.1). Three replicates were analyzed; significance was assessed by one-way ANOVA with Tukey’s test (GraphPad Prism 9). Primer sequences for BGC1–BGC5 and annealing temperatures used for each reaction are listed in Table A4. For standard-curve reactions, the template was 1 µL of amplicon (9–48 µg mL^−1^, diluted 0.5:10^3^ µL) plus 3.75 µL nuclease-free water.

## 5. Conclusions

Using newly formulated defined media, we revealed that carbon sources differentially regulate secondary metabolism in marine Bacteroidota. *Fulvivirga kasyanovii* 48LL grew on 31 of 34 substrates and, with 11 of them, produced antibacterial extracts, whereas *Tenacibaculum mesophilum* fLL utilized only 5 of 25 substrates. The whole genome sequencing of *F. kasyanovii* uncovered a 7.49 Mb genome harboring 16 BGCs, including a 570 kb island rich in hybrid NRPS-PKS BGCs. qPCR showed differential regulation of the BGCs within this region, where glucose upregulated BGC1, BGC3, and BGC4 while *κ*-carrageenan and PHB activated BGC2. BGC5 remained silent across all conditions. Importantly, standard yeast–peptone medium suppressed all active clusters. Carbon-source screening therefore, provides a practical route to unlock cryptic pathways in *F. kasyanovii* and potentially other marine Bacteroidota. Future work should focus on isolating and characterizing the metabolites produced under activating carbon sources and genetically linking them to their cognate BGCs.

## Data Availability

Data is contained within the present paper. *F. kasyanovii* 48LL genomic sequence has been deposited in the NCBI genome database under BioSample ID SAMN49894069.

## Abbreviations

The following abbreviations are used in this manuscript:

BGC	Biosynthetic gene cluster
NRPS	Non-ribosomal peptide synthase
PKS	Polyketide synthase
RiPPs	Ribosomally synthesized and post-translationally modified peptide
PHB	Polyhydroxybutyrate
qPCR	Quantitative polymerase chain reaction

## Appendix A

**Table A1:** Stock solutions for *T. mesophilum fLL* chemically defined media.

Reagent	Solution Concentration
L-glutamine	50 mg mL^−1^ HCl 1 M
L-histidine	50 mg mL^−1^ water
L-glutamic acid	50 mg mL^−1^ HCl 1 M
L-tyrosine	50 mg mL^−1^ HCl 1 M
L-cysteine hydrochloride	50 mg mL^−1^ water
L-methionine	50 mg mL^−1^ HCl 1 M
L-tryptophan	50 mg mL^−1^ HCl 0.5 M
trans-4-hydroxy-L-proline	50 mg mL^−1^ water
L-leucine	50 mg mL^−1^ HCl 1 M
L-phenylalanine	50 mg mL^−1^ KOH 0.5 M, heat, stir
L-valine	50 mg mL^−1^ water
L-aspartic acid	50 mg mL^−1^ HCl 0.5 M, heat, stir
L-asparagine	50 mg mL^−1^ HCl 1 M
L-threonine	50 mg mL^−1^ water
L-lysine hydrochloride	50 mg mL^−1^ water
L-serine	50 mg mL^−1^ water
L-arginine hydrochloride	50 mg mL^−1^ water
L-isoleucine	50 mg mL^−1^ KOH 0.5 M
L-alanine	50 mg mL^−1^ water
L-cystine	50 mg mL^−1^ HCl 1 M
Glycine	50 mg mL^−1^ water
Ferrous sulfate (FeII)	50 mg mL^−1^ water
Vitamin B12	0.5 mg mL^−1^ water

**Table A2:** Twenty-two amino acid solution for 100 mL 0.75× ASW (solutions from Table A1).

Amino Acid	Solution (µL)
Glycine	91.2
Arginine	91.2
Lysine	91.2
Cysteine	91.2
Glutamic acid	91.2
Hydroxyproline	91.2
Alanine	73
Proline	73
Aspartate	73
Serine	58
Leucine	29.2
Threonine	29.2
Valine	29.2
Phenylalanine	14.6
Isoleucine	14.6
Methionine	14.6
Histidine	7.3
Cystine	7.3
Tyrosine	7.3
Tryptophan	7.3
Asparagine	7.3
Glutamine	7.3

**Table A3:** Substrate description and their conditions used in defined media.

Substrate	Brand	Stock Solution	Sterilization	Amount per 1 mL Medium
Mango pectin	UNT Agroindustry Lab (Trujillo, Peru)	70 mg mL^−1^ water	Autoclave	14.2 µL
Food-grade pectin	Suman (Trujillo, Peru)	70 mg mL^−1^ water	Autoclave	14.2 µL
D(+) Glucose	Panreac (Barcelona, Spain)	200 mg mL^−1^ water	0.2 µm filter	20.0 µL
D(−) Fructose	Merck (Darmstadt, Germany)	100 mg mL^−1^ water	Autoclave	25.0 µL
Chitin	Spectrum (Middleton, WI, USA)	Used directly	Autoclave	0.400 mg
D(+) Xylose	Merck (Darmstadt, Germany)	20 mg mL^−1^ water	Autoclave	20.0 µL
L(−) Mannose	Spectrum (Middleton, WI, USA)	200 mg mL^−1^ water	0.2 µm filter	20.0 µL
Yeast extract (granulated)	Merck (Darmstadt, Germany)	Used directly	Autoclave	3.000 mg
Cold-water fish gelatin	Sigma (Burlington, MA, USA)	Used directly	Autoclave	3.000 mg
Dextran (Sephadex LH-20)	Sigma (Burlington, MA, USA)	Used directly	Autoclave	1.920 mg
λ-Carrageenan	ChemCruz (Huissen, The Netherlands)	10 mg mL^−1^ water	58 °C 2 h	150.0 µL
Xanthan gum	TCI (Tokyo, Japan)	5 mg mL^−1^ water	58 °C 2 h	300.0 µL
β-1,3-Glucan	ChemCruz (Huissen, The Netherlands)	20 mg mL^−1^ water	58 °C 2 h	40.0 µL
L(−) Fucose	ChemCruz (Huissen, The Netherlands)	20 mg mL^−1^ water	0.2 µm filter	200.0 µL
Carboxymethyl cellulose	Dropaksa (Trujillo, Peru)	25 mg mL^−1^ water	Autoclave	80.0 µL
Orange pectin	UNT Agroindustry Lab (Denton, TE, USA)	70 mg mL^−1^ water	Autoclave	14.2 µL
Polyhydroxybutyrate	UNT Environmental Eng. Lab (Denton, TE, USA)	Used directly	60 °C 24 h	0.260 mg
Glycerol (molecular biology grade)	Merck-Millipore (Darmstadt, Germany)	Used directly	Autoclave separately	20.0 µL
N-Acetyl-D-glucosamine	Thermo Fisher Scientific (Waltham, MA, USA)	50 mg mL^−1^ water	0.2 µm filter	40.0 µL
Fucoidan	Toronto Research Chemical (North York, Ontario. Canada)	20 mg mL^−1^ water	58 °C 2 h	25.0 µL
κ-Carrageenan	ChemCruz (Huissen, The Netherlands)	5 mg mL^−1^ water	58 °C 2 h	100.0 µL
Sodium alginate	Sigma (Burlington, MA, USA)	Used directly	Autoclave	2.000 mg
Corn-core xylan	TCI (Tokyo, Japan)	100 mg mL^−1^ water	58 °C 2 h	30.0 µL
L-(−) Galactose	TCI (Tokyo, Japan)	9.87 mg mL^−1^ water	0.2 µm filter	50.0 µL
N-Acetylneuraminic acid	ChemCruz (Huissen, The Netherlands)	49.45 mg mL^−1^ water	0.2 µm filter	40.0 µL
Starch	Sigma (Burlington, MA, USA)	Used directly	Autoclave	0.400 mg
Laminarin	TCI (Tokyo, Japan)	20 mg mL^−1^ water	0.2 µm filter	200.0 µL
D-Galactomannan	Alfa Aesar (Ward Hill, MA, USA)	16.6 mg mL^−1^ water	58 °C 2 h	25.0 µL
Glucuronic acid	Acros Organics (Waltham, MA, USA)	100 mg mL^−1^ water	0.2 µm filter	40.0 µL
Hyaluronic acid	TCI (Tokyo, Japan)	3 mg mL^−1^ water	58 °C 2 h	25.0 µL
L-Fructose	TCI (Tokyo, Japan)	20 mg mL^−1^ water	0.2 µm filter	25.0 µL
Heparin	Merck (Darmstadt, Germany)	12.5 mg mL^−1^ water	0.2 µm filter	40.0 µL
Chitosan	ChemCruz (Huissen, The Netherlands)	30 mg mL^−1^ water	58 °C 2 h	15.0 µL
D-Cellobiose	Sigma (Burlington, MA, USA)	65 mg mL^−1^ water	0.2 µm filter	7.5 µL

**Table A4:** Primers and qPCR conditions.

Target	Forward (5’→3’)	Reverse (5’→3’)	Amplicon (bp)	Annealing Temperature (°C)
BGC1	GACGGGTTGCAACTTAAGCCTTATAAG	GTTTAGTTCAAGAGAGATTCCCTCTGC	105	55
BGC2	GCGGTATGTGATATTTGGTGGCG	CACCGTTGTCTCGGTGATACC	108	59
BGC3	CATCATTCAGGAATACACCGAAGCTG	TTTCAATTGTCCGGCCGTTCTGG	103	59
BGC4	GCAGCTTCCTCAATATATGGTGCCTA	CCATTAATGGTAACAGGAAGGTGGC	69	55
BGC5	GCATGGTTTATTCAGGGACCGTTCA	CAAGCTCGGTCTGCTCTGTTCTA	112	56
16S rRNA	GTCAGTGGTGAAAGCCTGCAG	T GGTATCTATGCAT TTCACCGCTACAC	113	59

**Table A5:** Quality parameters obtained from qPCR standard curves.

Amplicon	% Efficiency	R^2^
BGC1	94.1	0.997
BGC2	91	0.984
BGC3	89.6	0.992
BGC4	101.6	0.992
BGC5	87.4	0.998
16S rRNA	91.9	0.996

**Table A6:** Predicted PUL substrates based on SusD sequence homology to DBCAN-PUL SusD entries. Substrates classified as “undetermined” have <80% coverage or <30% sequence identity.

Organism	Best SusD Match	Identity	Coverage	Predicted Substrate
*F. kasyanovii* 48LL 17 SusD genes in 10 PULs	*Zobellia galactanivorans* DsijT—63186 Pul43_Unidentified sulfated polysaccharide	55.98	96	Unidentified sulfated polysaccharide
	*Flavobacterium johnsoniae* UW101—376686Pul9_beta-glucan	37.87	97	Beta-glucan
	*Zobellia galactanivorans* DsijT—63186 Pul19_maltose	38.92	98	Maltose-containing polysaccharide
	*Bacteroides ovatus* ATCC 8483 411476Pul100_2_homogalacturonan	33.49	98	Homogalacturonan
	PUL0066_xyloglucan—*Bacteroides*	49.64	98	Xyloglucan
	PUL0164_beta-mannan—*Leeuwenhoekiella*	34.81	96	Beta-mannan
	*Flavobacterium johnsoniae* UW101—376686Pul10_starch-alpha-glucan	32.16	99	Starch, Alpha-glucan
	*Flavobacterium johnsoniae* UW101—376686Pul26_Pectin	56.96	99	Pectin
	*Gramella forsetii* KT0803—411154Pul3_beta-1,3-glucan laminarin	58.02	97	Beta-1,3-glucan, Laminarin
	*Zobellia galactanivorans* DsijT—63186Pul39	36.76	99	Sulfated polysaccharide
*T.mesophilum* Fll 11 SusD genes in 11 PULs	*Zobellia galactanivorans* DsijT—63186Pul22_unclear	72.4	100	Not determined
	PUL0554_dextran—*Bacteroides*	24.7	99	Undetermined
	PUL0547_mannose—*Bacteroides*	33.3	24	Undetermined
	*Capnocytophaga canimorsus* Cc5—860228Pul14_Iron uptake transferrin	36.9	99	Transferrin
	*Bacteroides fragilis* 638R-862962 Pul3_Nglycans	27	99	Undetermined
	*Zobellia galactanivorans* DsijT—63186 Pul48_mannose containing polysaccharide	32	52	Undetermined
	*Zobellia galactanivorans* DsijT—63186 Pul1_Xylan	38.3	66	Undetermined
	*Zobellia galactanivorans* DsijT—63186Pul29_alginate	24.2	97	Undetermined
	*Flavobacterium johnsoniae* UW101—376686 Pul10_starch-alpha-glucan	30.4	75	Starch
	*Flavobacterium johnsoniae* UW101—376686Pul34_2_chitin	28.8	98	Undetermined
	*Flavobacterium johnsoniae* UW101—376686Pul21_peptide	29.9	97	Undetermined
